# Clinical and surgical factors and intraoperative complications in patients who underwent penetrating keratoplasty

**DOI:** 10.1590/1518-8345.2733-3141

**Published:** 2019-04-29

**Authors:** Giovanna Karinny Pereira Cruz, Marcos Antonio Ferreira, Isabelle Campos de Azevedo, Viviane Euzébia Pereira Santos, Vanessa Giavarotti Taboza Flores, Elenilda de Andrade Pereira Gonçalves

**Affiliations:** 1 Universidade Federal do Rio Grande do Norte, Natal, RN, Brasil.; Bolsista da Coordenação de Aperfeiçoamento de Pessoal de Nível Superior (CAPES), Brasil.; 2 Universidade Federal do Mato Grosso do Sul, Departamento de Enfermagem, Campo Grande, MS, Brasil.; 3 Universidade Federal do Rio Grande do Norte, Natal, RN, Brasil.; Bolsista do Conselho Nacional de Desenvolvimento Científico e Tecnológico (CNPq), Brasil.; 4 Universidade Federal do Rio Grande do Norte, Departamento de Enfermagem, Natal, RN, Brasil.; 5 Universidade Federal do Mato Grosso do Sul, Hospital Universitário Maria Aparecida Pedrossian, Campo Grande, MS, Brasil.; 6 Universidade Católica Dom Bosco, Campo Grande, MS, Brasil.

**Keywords:** Eye, Cornea, Keratoplasty, Penetrating, Intraoperative Complications, Corneal Transplantation, Cataract Extraction, Olho, Córnea, Ceratoplastia Penetrante, Complicações Intraoperatórias, Transplante de Córnea, Extração de Catarata, Ojo, Córnea, Ceratoplastia Penetrante, Complicaciones Intraoperatorias, Trasplante de Cornea, Extracción de Catarata

## Abstract

**Objective:**

to identify the main intraoperative complications of patients who underwent keratoplasty and relationship between these complications and clinical and surgical factors.

**Method:**

cross-sectional observational study. A census of the patients submitted to keratoplasty was carried out, which totaled 258 procedures.

**Results:**

twenty-two intraoperative complications were recorded, all in penetrating keratoplasty surgeries, of which 59.09% were performed in male patients with a mean age of 58.5 years. The main intraoperative complication was vitreous loss (36.36%). A statistically significant relationship was found between the variable “intraoperative complication” and the variables “previous surgery”, “combined keratoplasty and cataract extraction” and “corneal host button greater than 8.0 mm”.

**Conclusion:**

identifying the main intraoperative complications of keratoplasty enables nurses to understand which factors may interfere with these procedures, point out possible predictors of complications, and seek control measures so that such complications do not occur.

## Abstract (pt)

**Objetivo:**

identificar as principais complicações intraoperatórias dos pacientes que realizaram ceratoplastias e sua relação com fatores clínicos e cirúrgicos.

**Método:**

estudo transversal, observacional. Realizou-se um censo dos pacientes submetidos a ceratoplastias que totalizou 258 procedimentos.

**Resultados:**

foram registradas 22 complicações intraoperatórias, todas em ceratoplastias penetrantes. Do total, 59,09% foram realizadas em pacientes do sexo masculino com idade média de 58,5 anos. A principal complicação intraoperatória notificada foi a perda vítrea (36,36%). Encontrou-se relação estatisticamente significativa entre a variável “complicação intraoperatória” e as variáveis “cirurgia prévia”, “ceratoplastia combinada com extração de catarata” e “botão corneano do receptor maior que 8,0 mm”.

**Conclusão:**

identificar as principais complicações intraoperatórias da ceratoplastia possibilita à enfermagem compreender quais fatores podem interferir nesses procedimentos, apontar possíveis fatores preditores das complicações e buscar medidas de controles para que tais complicações não ocorram.

## Abstract (es)

**Objetivo:**

identificar las principales complicaciones intraoperatorias de los pacientes que realizaron queratoplastias y su relación con factores clínicos y quirúrgicos.

**Método:**

estudio transversal, observacional. Se realizó un censo de los pacientes sometidos a las queratoplastias que tuvo un total de 258 procedimientos.

**Resultados:**

fueron registradas 22 complicaciones intraoperatorias, todas en queratoplastias penetrantes. Del total, 59,09% fueron realizadas en pacientes del sexo masculino con edad media de 58,5 años. La principal complicación intraoperatoria notificada fue la pérdida vítrea (36,36%). Fue encontrada relación estadísticamente significativa entre la variable “complicación intraoperatoria” y las variables “cirugía previa”, “queratoplastia combinada con extracción de catarata” y “botón corneal del receptor mayor que 8,0 mm”.

**Conclusión:**

identificar las principales complicaciones intraoperatorias de la queratoplastia posibilita a la enfermería comprender cuáles factores pueden interferir en estos procedimientos, apuntar posibles factores predictores de las complicaciones y buscar medidas de controles para que tales complicaciones no ocurran.

## Introduction

Corneal transplantation is primarily aimed at visual rehabilitation. The procedure itself can often cause refractive abnormality, such as high degrees of astigmatism, irregularity or anisometropia, which may hinder the restoration of satisfactory vision^(^ ^1^ ^)^.

With the evolution of corneal transplantation techniques, more lamellar surgeries have been performed around the world and the safety of transplantation has increased. In addition to other advantages, lamellar surgery has shown fewer complications, since the integrity of the patient’s globe is preserved^(^ ^2^ ^)^.

Penetrating keratoplasty is considered a successful intraocular procedure, with a high success rate in low-risk corneal diseases. It can be performed under general or local anesthesia. There are, however, intraoperative complications of keratoplasties that can seriously impair vision, cause rejection episodes and/or even graft failure^(^ ^1^ ^-^ ^3^ ^)^.

According to the American Academy of Ophthalmology, the main intraoperative complications in keratoplasty refer to graft centralization, irregular trepanation, damage to lens, damage to donor tissue, choroidal bleeding and effusion, and incarceration of the iris and vitreous tissue in the anterior chamber. Although the literature shows the main intraoperative complications during a keratoplasty, there is no current data on the epidemiological profile of the subjects exposed to these complications. However, the monitoring and prophylaxis of complications during keratoplasty includes elements involved in the preoperative and intraoperative periods^(^ ^4^ ^-^ ^5^ ^)^.

The nursing appointment is an important tool for the investigation and implementation of care that guarantees to the patient the ideal conditions for performing the transplantation and maintenance of the graft in the postoperative period. In the state of Rio Grande do Norte, the follow-up of these patients from the preoperative to the postoperative period is performed by the medical ophthalmologic team, while the nursing team acts during intraoperative care^(^ ^6^ ^-^ ^7^ ^)^.

Nurses’ performance must cover all surgical periods, from the indication to the transplantation to the patient’s discharge. The nursing appointment enables identifying risk factors, comorbidities, therapeutic adherence, adequate use of medications, physical ophthalmologic examination, and control of modifiable risk factors and, consequently, improving graft quality and transparency for a longer time and avoiding possible complications^(^ ^7^ ^)^.

In view of the difficulty of identifying of the main intraoperative complications and their possible causes, this study aims to identify the main intraoperative complications of the patients who performed keratoplasty and the relation of these complications with clinical and surgical factors.

## Method

This is a quantitative, epidemiological, observational, cross-sectional study carried out at a university hospital of Natal, Brazil, which is a public reference in the performance of keratoplasty.

The census sample was composed of the keratoplasties performed between 2010 and 2014. This period was chosen because 2010 was the year in which keratoplasty began to be performed in the said university hospital and 2014 was the previous year to the data collection of the present study, resulting in a five-year period. The analysis included 258 keratoplasties that met the eligibility criteria, namely keratoplasties performed in individuals of all ages and of both sexes followed-up by the service during the studied period, regardless of the clinical condition indicative for the procedure.

The data collection was carried out based on the documentary records of the hospital service after the survey of transplanted patients in that period, using a structured form developed specifically for this study in order to systematize the collection of data necessary to meet the proposed objectives.

The structured form was designed to investigate clinical and surgical variables, namely sex, age, operated eye, glaucoma, previous surgery, vascularization, eye classification, type of surgery, type of keratoplasty, donor corneal button size, recipient corneal button size, keratoplasty combined with cataract extraction, suture technique, and time between tissue preservation and transplantation. The form contained closed questions that were answered using the data available in the service database.

Data were processed and analyzed using Statistical Package for Social Sciences (SPSS), version 20.0, and presented in tables. Descriptive statistics was used for univariate analysis using absolute and relative frequency and mean. For inferential analysis between the variable “intraoperative complications” and the variables “gender, age, operated eye, glaucoma, previous surgery, vascularization, eye classification, type of surgery, type of keratoplasty, donor button size, host button size, combination with cataract extraction, suture technique and time interval between tissue preservation and transplantation”, the Chi-square (X^2^) or Fisher’s exact tests were used. The significance level was set at 0.05.

The research protocol was approved by the Research Ethics Committee of the Federal University of Rio Grande do Norte in its ethical and methodological aspects, according to resolution CNS no. 466/2012, under opinion 876.177 and CAAE no. 37533014.8.0000.5537.

## Results

During the period from January 2010 to December 2014, 258 keratoplasties were performed in the analyzed service, of which 22 (8.53%) intraoperative complications were recorded. Al complications (100%) occurred in penetrating keratoplasties, being 59.09% in male patients and in right eyes. The mean age of patients with intraoperative complications was 58.5 years, with a minimum of 18 and a maximum of 90 years.

The main intraoperative complications were vitreous loss (36.36%), followed by expulsion of intraocular/crystalline lens (13.64%), vitreous hypertension (9.09%) and bleeding (9.09%).

Table 1 presents the bivariate analysis of the variable “intraoperative complications” with the clinical and surgical characteristics of patients submitted to keratoplasty.

**Table 1:** – Intraoperative complications *versus* clinical and surgical characteristics in penetrating keratoplasty (n=258). Natal, RN, Brazil, 2015

Characteristic	Intraoperative complication		Total	p*

	Yes n (%)	No n (%)		
Sex				
Male	13 (10.66)	109 (89.34)	122	0.405^†^
Female	09 (7.56)	110 (92.44)	119	
Age				
Up to 20 years	01 (3.70)	26 (96.30)	27	0.058^†^
21 - 30 years	01 (2.27)	43 (97.73)	44	
31 - 40 years	00 (0.00)	23 (100.0)	23	
41 - 50 years	05 (19.23)	21 (80.77)	26	
51 - 60 years	04 (10.81)	33 (89.19)	37	
More than 60 years	11 (13.10)	73 (86.90)	84	
Glaucoma				
Yes	04 (16.00)	21 (84.00)	25	0.208^†^
No	18 (8.33)	198 (91.67)	216	
Previous surgery				
Yes	13 (13.83)	81 (86.17)	94	0.043^†^
No	09 (6.12)	138 (93.88)	147	
Vascularization				
Yes	13 (12.87)	88 (87.13)	101	0.087^†^
No	09 (6.43)	131 (93.57)	140	
Eye classification				
Phakic	14 (7.65)	169 (92.35)	183	0.290^†^
Pseudophakic	07 (13.73)	44 (86.27)	51	
Aphakic	01 (20.00)	04 (80.00)	05	
Type of surgery				
Elective	12 (6.94)	161 (93.06)	173	0.059^†^
Urgency	10 (14.71)	58 (85.29)	88	
Type of keratoplasty				
Penetrating	22 (9.82)	202 (90.18)	224	0.378^‡^
Lamellar	00 (0.00)	17 (100.0)	17	
Donor button size				
Up to 8.4	04 (5.63)	67 (94.37)	71	0.223^†^
More than 8.4	18 (10.59)	152 (89.41)	170	
Host button size				
Up to 8.0	12 (5.97)	189 (94.03)	201	0.001^‡^
More than 8.0	10 (25.00)	30 (75.00)	40	
Combined with cataract extraction				
Yes	06 (35.29)	11 (64.71)	17	0.002^‡^
No	16 (7.14)	208 (92.86)	224	
Suture technique				
Continuous	00 (0.00)	02 (100.0)	02	0.696^†^
Interrupted	22 (9.40)	212 (90.60)	234	
Combined	00 (0.00)	05 (100.0)	05	
Time between tissue preservation and transplantation				
Up to 10 days	13 (10.16)	115 (89.84)	128	0.434^†^
More than 10 days	08 (7.27)	102 (92.73)	110	

*p-value; †Chi-square test; ‡Fisher’s exact test

Statistically significant differences were found between the variable “intraoperative complications” and “previous surgery”, “host button size” and “combination with cataract extraction” using the chi-square test (X^2^) or Fisher’s exact test, at a significance level of 5%. Intraoperative complications were more prevalent in patients who had undergone a previous surgery, with host button size above 8.0 mm, and when the surgery was combined with cataract extraction.

Patients who had undergone previous surgery had 2.46 times more intraoperative complications than those who had not undergone such procedures.

Patients with a host button above 8.0 mm had 5.26 times more intraoperative complications than those with a host button less than or equal to 8.0 mm.

When keratoplasty was combined with cataract extraction, it had 7.09 times more complications when compared to keratoplasty performed alone.

Table 2 presents the prevalence ratio of the variables “previous surgery”, “host button size” and “combination with cataract extraction” *versus* the presence of “intraoperative complications”.

**Table 2:** Prevalence ratio of intraoperative complications and surgical variables with statistical significance. Natal, RN, Brazil, 2015

Variable	Prevalence Ratio	Confidence Interval (95%)	

		Lower	Higher
Previous surgery	2.46	1.01	6.01
Host button	5.26	2.08	13.16
Combined with cataract extraction	7.09	2.32	21.67

## Discussion

In the present study, all intraoperative complications in keratoplasty occurred in penetrating surgeries. Because it is an intraocular procedure, conventional penetrating keratoplasty has surgical risks, particularly during the time the anterior chamber is exposed in the open air. Risks include expulsive choroidal bleeding, positive vitreous pressure that can lead to lens expulsion, iris sphincter trauma and/or vitreous loss and endophthalmitis. These are the most serious possible complications of penetrating keratoplasties when compared to anterior and endothelial lamellar keratoplasties^(^ ^2^ ^-^ ^3^ ^,^ ^8^ ^)^.

New positive results were achieved with the adoption of the deep anterior lamellar keratoplasty (DALK). Because it is an extraocular procedure, it presents important safety and survival advantages of the corneal endothelium^(^ ^9^ ^)^. However, penetrating keratoplasty is still performed by many surgeons and the prevention of the serious complications deriving from this procedure is of great interest to all who promote eye health^(^ ^2^ ^)^.

In this study, the main intraoperative complication of penetrating keratoplasty was the vitreous loss (36.36%). Vitreous loss is an intraoperative complication that occurs in high-risk penetrating keratoplasty because this is a procedure in which the anterior chamber is exposed to open air^(^ ^8^ ^)^.

Positive posterior pressure or positive vitreous pressure during penetrating keratoplasty is a high-risk eye complication that can lead to vitreous loss, especially if followed by choroidal bleeding. A study in Croatia stated that positive posterior pressure occurred in 3.6% of the cases, whereas in the present study it occurred in 0.78% of the keratoplasties performed. Because it is a complication that can lead to loss of vision, it is important to identify surgical mechanisms and techniques that may prevent more intraoperative complications without damaging the donated tissue^(^ ^2^ ^)^.

As a solution to this intraoperative complication, some studies propose innovative surgical techniques that promote intraoperative safety of the anterior chamber and consequently reduce the risk of vitreous complications^(^ ^8^ ^)^. The graft-over-host technique aims to overcome positive vitreous pressure during penetrating keratoplasty as an alternative to minimize anterior chamber exposure. The tecnique deals with a type of adapted penetrating keratoplasty, whose graft of the donor is initially superimposed on that of the host and only later this latter is removed^(^ ^2^ ^)^.

The inferential analysis of the variable “intraoperative complications” with clinical and surgical variables found a statistically significant association in relation to “previous surgery”, “host button size above 8.0 mm” and “combination with cataract extraction”.

Patients had performed some kind of previous ophthalmologic surgery in 59.09% of the keratoplasties with intraoperative complications. Facectomy represented 61.54% of these previous surgeries, being 87.5% in pseudophakic eyes (with intraocular lenses) and 12.5% in aphakic eyes (without the use of intraocular lenses).

A study conducted in Turkey shows that previous surgeries such as vitrectomy and iridectomy may be associated with intraoperative complications. Other factors evidenced by the study as possible predictive factors for intraoperative complications were coexisting ocular pathology and the level of professional experience of surgeons in performing keratoplasty^(^ ^3^ ^)^.

The prevalence ratio of 2.46 times more intraoperative complications in patients submitted to previous surgeries may be related to complications and tissue damage caused by previous surgical procedures, such as endophthalmitis, commonly associated with facectomies^(^ ^10^ ^-^ ^11^ ^)^.

The accomplishment of keratoplasty combined with cataract extraction (facectomy) presented, in this study, a statistical significance when correlated with the presence of intraoperative complications, with a prevalence ratio of 7.09 times. However, this relationship presents divergences in the literature. This is because some studies point to the performance of combined surgeries as something that can bring about intraoperative complications and future damage to ocular health, but others relate the combined technique with positive and less cost-effective results once it does not expose the patient to two procedures at different times and presents a good ocular prognosis^(^ ^12^ ^-^ ^13^ ^)^.

A study carried out in Saudi Arabia aimed to evaluate the results of surgeries of corneal grafts in which patients had undergone cataract surgery simultaneous to penetrating keratoplasty. As a result, the study presented evidence that the accomplishment of a combined procedure results in a faster visual rehabilitation and a graft with good clarity^(^ ^12^ ^)^.

In Japan, the Tohoku Graduate School of Medicine presented a surgical technique called Chandelier Illumination for performing keratoplasty surgery combined with cataract extraction. It is a technique in which the anterior chamber is not exposed, which minimizes intraoperative and postoperative complications. The rate of successful surgeries was significantly higher in the group that used the Chandelier technique than in the non-Chandelier group, with rates of 86% and 30%, respectively^(^ ^13^ ^)^.

Literature shows that the use of a corneal button 0.25-0.50 mm larger than the host’s diameter should be recommended for preventing and reducing corneal excessive flattening in the postoperative period, and for reducing secondary glaucoma and improving conditions for wound closure^(^ ^4^ ^,^ ^14^ ^)^. However, the association of the “intraoperative complications” with the “host corneal buttons over 8.0 mm” presented a prevalence ratio 5.16 times greater than corneal buttons smaller or equal to 8.00 mm. This data should be taken into account by future longitudinal studies, since there are no more studies that report this association and verify the relationship between donor-host button size differences and intraoperative complications.

The findings of this study showed that, in addition to routine postoperative follow-up, the identification and prophylaxis of complications in penetrating keratoplasty include preoperative and intraoperative nursing care. Preoperative prophylaxis consists in the treatment of systemic diseases and eyelid abnormalities, in determining the size of the corneal graft, in avoiding penetrating keratoplasty in cases of uncontrolled intraocular pressure, in avoiding penetrating keratoplasty in cases of corneal hydrops, in providing the preoperative treatment in cases of vascularized cornea, amniotic membrane transplantation prior to penetrating keratoplastic with ulcerative keratitis, in addition to ensuring a better quality control of the transplantations and preoperative counseling that results in greater adherence to the treatment^(^ ^1^ ^,^ ^5^ ^)^.

Intraoperative prophylaxis encompasses the control of hypotension and complete relaxation during general anesthesia, prevention of decentralization, horizontal torsion and vertical inclination using a noncontact trephination technique (preferably excimer laser), with double cross stitch sutures and continuous and application of Flieringa rings in vitrectomized aphakic eyes. In the postoperative period, periodic exams using fluorescein and blue light are indispensable. All loose sutures have to be removed as soon as possible. In cases of herpetic disease, antivirals should be given. In cases of epithelial defects, therapy with autologous serum dropper or amniotic membrane patches are valid options. Immune reactions should be diagnosed and treated immediately^(^ ^5^ ^)^.

The necessary care for the prevention and control of complications in keratoplasty includes attention and multiprofessional management. During the appointments, the nursing team should be attentive to the identification of risk factors for complications in keratoplasty, management of exposed patients, and prevention of modifiable risk factors.

Since it is a documentary research whose source of data collection originated from secondary data, like any study that uses this technique, it may have some limiting factors, such as loss of important information, inaccuracy of data, and the weaknesses of information systems records.

Another limiting factor of this study is the cross-sectional design. Therefore, longitudinal studies could be performed in order to identify the relationship of the variables whose statistical analysis inferred association.

## Conclusion

The present study verified that vitreous loss was the main intraoperative complication in keratoplasties, and that surgical factors such as previous ocular surgery, host corneal button size greater than 8 mm, and keratoplasty combined with cataract extraction were related to the presence of intraoperative complications.

The prevention and identification of the main intraoperative complications compose the nursing care to patients who will undergo keratoplasty. For the adequate management of these patients, the nursing care should follow the entire perioperative period, since it may help preventing modifiable risk factors and provide adequate management of the non-modifiable risk factors.

Therefore, preventive mechanisms should be used for these complications, such as the use of new surgical procedures that minimize such damages, as well as multidisciplinary care that guarantees continued care to the patient from the preoperative and intraoperative period until the postoperative period.
